# Axonal Protection by Nicotinamide Riboside via SIRT1-Autophagy Pathway in TNF-Induced Optic Nerve Degeneration

**DOI:** 10.1007/s12035-020-02063-5

**Published:** 2020-08-20

**Authors:** Yasushi Kitaoka, Kana Sase, Chihiro Tsukahara, Naoki Fujita, Ibuki Arizono, Hitoshi Takagi

**Affiliations:** 1 Department of Molecular Neuroscience, St. Marianna University Graduate School of Medicine, 2-16-1 Sugao, Miyamae-ku, Kaswasaki, Kanagawa 216-8511 Japan; 2 Department of Ophthalmology, St. Marianna University School of Medicine, Kawasaki, Japan

**Keywords:** Nicotinamide riboside, NRK1, Autophagy, SIRT1, p62

## Abstract

Nicotinamide adenine dinucleotide (NAD^+^) synthesis pathway has been involved in many biological functions. Nicotinamide riboside (NR) is widely used as an NAD^+^ precursor and known to increase NAD^+^ level in several tissues. The present study aimed to examine the effect of NR on tumor necrosis factor (TNF)-induced optic nerve degeneration and to investigate whether it alters SIRT1 expression and autophagic status in optic nerve. We also examined the localization of nicotinamide riboside kinase 1 (NRK1), which is a downstream enzyme for NR biosynthesis pathway in retina and optic nerve. Intravitreal injection of TNF or TNF plus NR was performed on rats. The p62 and LC3-II protein levels were examined to evaluate autophagic flux in optic nerve. Immunohistochemical analysis was performed to localize NRK1 expression. Morphometric analysis showed substantial axonal protection by NR against TNF-induced axon loss. TNF-induced increment of p62 protein level was significantly inhibited by NR administration. NR administration alone significantly increased the LC3-II levels and reduced p62 levels compared with the basal levels, and upregulated SIRT1 levels in optic nerve. Immunohistochemical analysis showed that NRK1 exists in retinal ganglion cells (RGCs) and nerve fibers in retina and optic nerve. NR administration apparently upregulated NRK1 levels in the TNF-treated eyes as well as the control eyes. Pre-injection of an SIRT1 inhibitor resulted in a significant increase of p62 levels in the NR plus TNF treatment group, implicating that SIRT1 regulates autophagy status. In conclusion, NRK1 exists in RGCs and optic nerve axons. NR exerted protection against axon loss induced by TNF with possible involvement of upregulated NRK1 and SIRT1-autophagy pathway.

**Electronic supplementary material:**

The online version of this article (10.1007/s12035-020-02063-5) contains supplementary material, which is available to authorized users.

## Introduction

Nicotinamide adenine dinucleotide (NAD^+^) synthesis pathway has been involved in many biological functions. NAD^+^ is synthesized by salvage of vitamin precursors, nicotinic acid (NA), nicotinamide, and nicotinamide riboside (NR). Nicotinamide phosphoribosyltransferase (Nampt) converts nicotinamide into nicotinamide mononucleotide (NMN), whereas nicotinamide riboside kinase 1 (NRK1) converts NR into NMN. Then, NMN is converted to NAD^+^ by nicotinamide nucleotide adenylyltransferase1–3 (Nmnat1–3) [1]. Nmnat1–3 are enzymes which have been reported to link to axonal protection in dorsal root ganglia [2–6]. Nmnat1–3 were found to exist in optic nerve [7–9], and Nmnat2 is required to retinal ganglion cell (RGC) axon growth [9]. It was reported that cytoplasmic overexpression of Nmnat1 protected against glaucomatous RGC axon loss [10]. Our previous study demonstrated that overexpression of Nmnat3 protected against glaucomatous RGC axon loss and tumor necrosis factor (TNF)-induced axon loss [8]. On the other hand, a previous study showed a decrease in NAD level in retina in DBA/2J mice [11]. This is agreement with our previous study showing a decrease in NAD level in optic nerve in TNF-induced axon damage model [7]. Interestingly, a recent study demonstrated a significantly lower plasma nicotinamide concentration in primary open-angle glaucoma patients compared with the control group [12]. Since oral intake of vitamin B3/nicotinamide increased NAD level in retina and exerted axonal protection in DBA/2 J mice [11], it is reasonable to postulate that the nicotinamide supplementation may have a beneficial effect for certain glaucomatous damages.

NR is widely used as an NAD^+^ precursor supplementation and has been reported to increase the blood NAD^+^ level in humans [13]. NRK1 is the central rate-limiting enzyme in driving NAD^+^ synthesis from NR [14]. However, localizations of NRK1 in retina and optic nerve have not been documented. There have been several studies suggesting critical roles of NAD^+^ and its precursors on autophagy machinery [15–17]. Autophagy is a cellular process including the clearance of unnecessary proteins and maintains homeostasis in several types of neurons. We and others reported that autophagy plays crucial roles in certain different optic nerve damages such as optic nerve crush model, hypertensive glaucoma model, and TNF-induced axon damage model [18–21]. For autophagy research, SQSTM1/p62 is used as a marker and decrease of p62 level is associated with autophagy activation [22]. In yeast, NR upregulates NAD^+^ levels, enhances Sir2 functions, and extends lifespan [23]. Sirtuin 1 (SIRT1), the mammalian homolog of yeast Sir2, can be activated by calorie restriction [24], NAD^+^, and its precursors [25]. Thus, the purpose of present study is to examine the effect of NR on TNF-induced axonal degeneration and to investigate whether it alters SIRT1 expression and autophagic status in optic nerve. We also examined the localization of NRK1, which is a downstream enzyme for NR biosynthesis pathway in retina and optic nerve as well as the alteration of NRK1 expression. Finally, we tested if an inhibitor of SIRT1 alters autophagy status.

## Materials and Methods

### Animals

Experiments were carried out on 8-week-old male Wistar rats. All studies were conducted according to the ARVO Statement for the Use of Animals in Ophthalmic and Vision Research and approved by Ethics Committee of the Institute of Experimental Animals of St. Marianna University School of Medicine. The animals were kept in the controlled rooms (23 ± 1 °C; humidity at 55 ± 5%; light from 6 a.m. to 6 p.m.).

### Intravitreal Administrations

Intravitreal injection of TNF (Sigma-Aldrich, St. Lois, MO) was performed as described previously [7]. Phosphate-buffered saline (PBS) was used as a control. Anesthetization with intramuscular injections of a mixture of ketamine-xylazine was conducted. NR triflate was purchased from Toronto Research Chemicals (North York, ON, Canada), dissolved in PBS. Concomitant injection of 2, 20, and 200 pmol of NR and 10 ng TNF was performed intravitreally. For immunoblotting, NR alone injection was also performed. For the SIRT1 inhibitor study, EX-527 (Sigma-Aldrich) was dissolved in DMSO and 200 pmol of EX-527 or DMSO alone was injected intravitreally 10 min before intravitreal injection of NR plus TNF. One and 2 weeks after intravitreal injection, the rats were euthanatized with overdose of sodium pentobarbital and the eyes were enucleated.

### Immunoblotting

Optic nerve specimens (4-mm lengths) were gathered and homogenized in protein extraction buffer 1 week after injection. Homogenized samples were then centrifuged at 15,000×g for 15 min at 4 °C. Protein concentrations were determined with the supernatants. Each sample (3 μg) was applied and subjected to the mini gel (Bio-Rad Laboratories) and transferred to enhanced chemiluminescent membrane (EMD Millipore Corporation, Temecula, CA). The membranes were blocked with 5% skim milk with tris buffered saline (TBS) containing Tween-20 and reacted with anti-p62 antibody (MBL Life Science, Nagoya, Japan), anti-LC3 antibody (MBL Life Science), anti-SIRT1 antibody (Santa Cruz Biotechnology), anti-NRK1 antibody (Lifespan Biosciences Inc. Seattle, WA) or anti-β-actin antibody (Sigma-Aldrich). After three times washing, the membranes were reacted with anti-rabbit or anti-mouse peroxidase-labeled secondary antibody (MP Biochemicals, Solo, OH). Immunoblotting was visualized with a chemiluminescence detection system (ECL Plus Western Blotting Detection Reagents, Amersham Pharmacia Biotech).

### Immunohistochemistry

Three eyes 1 week after intravitreal injection of NR or three normal eyes were collected and fixed by immersion in 4% paraformaldehyde, dehydrated, and embedded in paraffin. Sections were made through the optic disc and blocked with 1% bovine serum (Roche Diagnostics GmbH, Mannheim, Germany). The primary antibodies were against NRK1 (1:100; LifeSpan BioSciences), neurofilament-L (a marker of nerve fibers; 1:100; Dako, Tokyo, Japan), or Thy-1 (a marker of RGC; 1:50; Santa Cruz Biotechnology, TX). The secondary antibodies were FITC-labeled or rhodamine-labeled antibodies (1:100; Cappel, Aurora, OH). The sections were mounted on slides in DAPI-containing medium with cover glass.

### Quantification of Optic Nerve Axons

Optic nerve specimens (4-mm lengths from 1 mm behind the globe) were collected and soaked in Karnovsky’s solution for 24 h at 4 °C 2 weeks after injection. Several dehydrations were performed, and samples were embedded in acrylic resin at 70 °C two overnight. Then, samples were sectioned and stained with 1% paraphenylene-diamine (Sigma-Aldrich) in absolute methanol [7, 26]. This can stain myelin, and five black and white images from each eye were obtained at the center and at each quadrant of the periphery with a light microscope (Olympus, Tokyo, Japan). These black and white images (each area is 5850 μm^2^, and total area is 29,250 μm^2^ per eye) were used for quantification with the Aphelion image processing software (ADCIS S.A., Hérouville Saint-Clair, France). The number of axons was averaged in each eye and each group, and data were presented as the number per square millimeter. After quantification, representative color photos were obtained.

### Statistical Analysis

Data are expressed as mean ± SEM. Differences among groups were analyzed by one-way ANOVA with post-hoc Tukey’s HSD test or Mann–Whitney method. A probability value was considered statistically significant when *p* < 0.05.

## Results

### Effects of NR on TNF-Induced Axon Loss in Optic Nerve

**Fig. 1:**
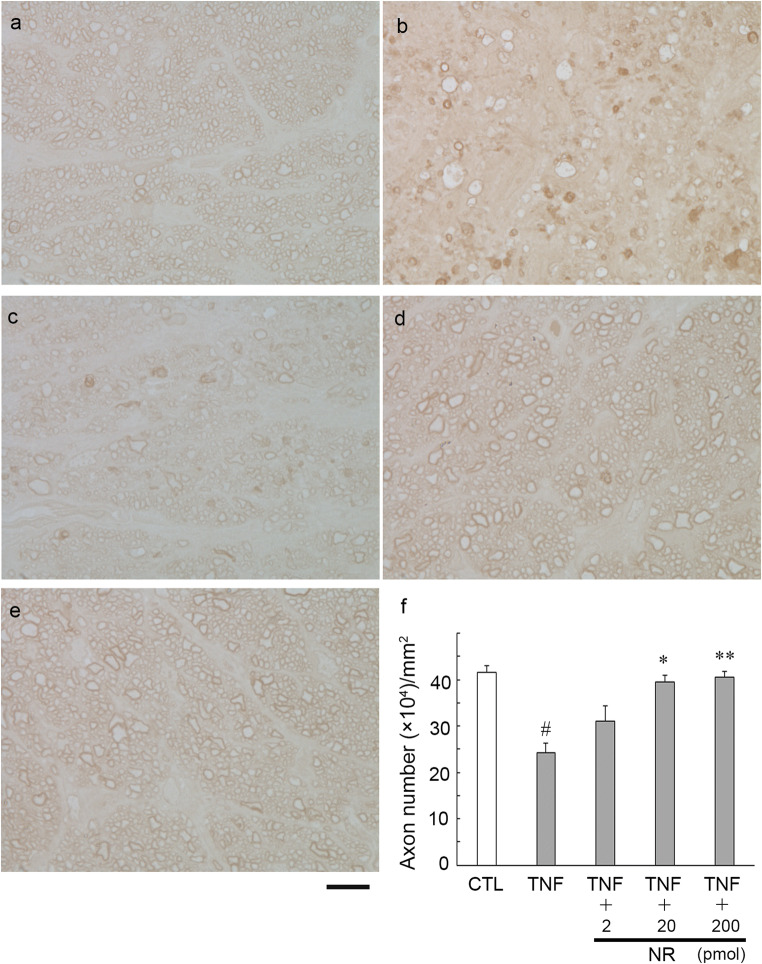
Paraphenylene-diamine staining of optic nerve axons 2 weeks after injection. **a** Control group. **b** TNF-injected group. **c** 2 pmol NR + TNF-injected group. **d** 20 pmol NR + TNF-injected group. **e** 200 pmol NR + TNF-injected group. Scale bar = 10 μm. (f) Morphometric analysis of axon number. (CTL: *n* = 5, TNF: *n* = 5, 2 pmol NR + TNF: *n* = 6, 20 pmol NR + TNF: *n* = 7, 200 pmol NR + TNF: *n* = 7) (*P*^*#*^ < 0.0001 vs. CTL, P^*^ < 0.0005 vs. TNF, P^**^ < 0.0001 vs. TNF)

As shown previously [7], histological findings again showed substantial degenerative changes and apparent axon losses after TNF injection (Fig. 1b) compared with the control (Fig. 1a). Co-injection with 2 pmol NR plus TNF showed slightly protective tendency (Fig. 1c). However, this was not statistically significant (*p* = 0.1850 vs. TNF; Fig. 1f). Co-injection with 20 or 200 pmol NR plus TNF showed noticeable protective effects compared with the TNF alone injection (Fig. 1d and e, respectively). The quantitative analysis showed remarkable protective effects against TNF-induced axon loss, and these were statistically significant (20 pmol and 200 pmol NR: *p* = 0.0002 vs. TNF, and *p* < 0.0001 vs. TNF, respectively; Fig. 1f).

### Effects of TNF and NR on LC3-II Protein Levels in Optic Nerve

**Fig. 2:**
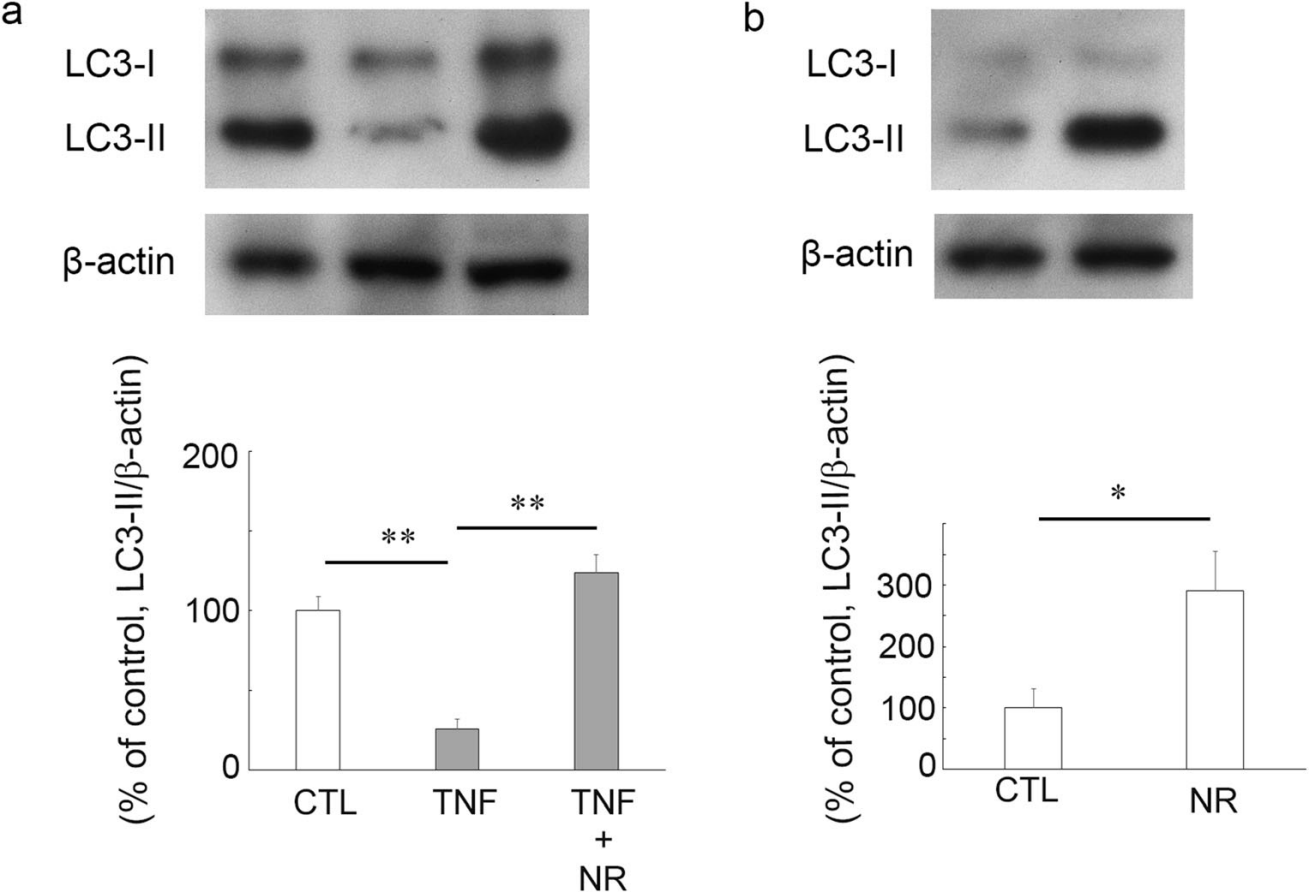
Immunoblotting in samples from optic nerves 1 week after injection. a Effects of TNF or 200 pmol NR + TNF on LC3-II protein level (CTL: *n* = 3, TNF: *n* = 3, TNF + NR: *n* = 3) (P** < 0.005). (b) Effects of 200 pmol NR on LC3-II protein level. (CTL: *n* = 3, NR: n = 3) (P* < 0.05)

There was a significant increase in the LC3-II level in the treatment with 200 pmol NR plus TNF as compared with those in the treatment with TNF at 1 week (Fig. 2a). Moreover, 200 pmol NR alone administration significantly increased the LC3-II level compared with the control group (Fig. 2b).

### Effects of TNF and NR on p62 Protein Levels in Optic Nerve

**Fig. 3:**
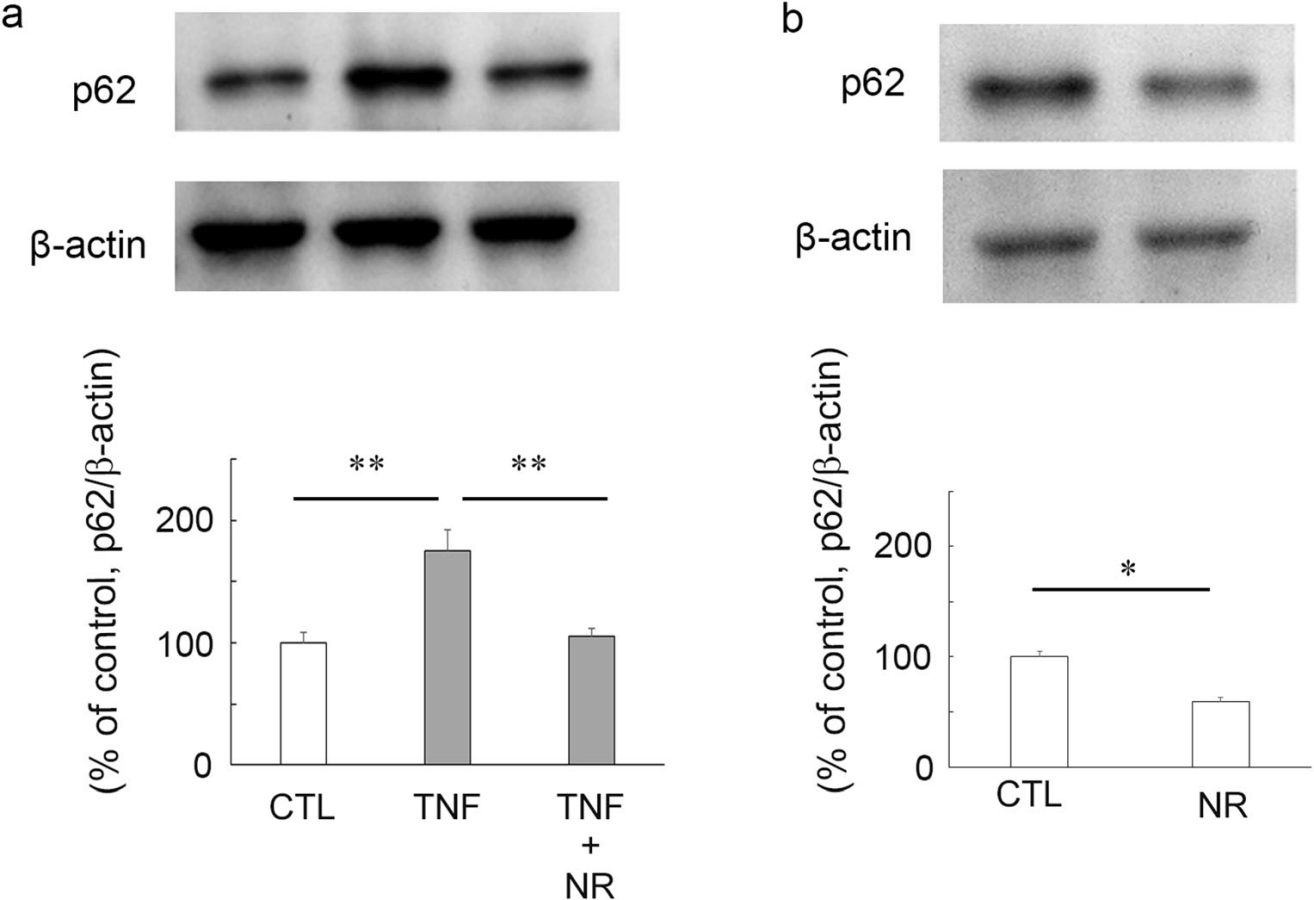
Immunoblotting in samples from optic nerves 1 week after injection. **a** Effects of TNF or 200 pmol NR + TNF on p62 protein level. (CTL: *n* = 4, TNF: *n* = 4, TNF + NR: *n* = 4) (P** < 0.005). **b** Effects of 200 pmol NR on p62 protein level. (CTL: *n* = 5, NR: *n* = 5) (P* < 0.05)

In agreement with our previous findings [27], p62 protein level was significantly increased in optic nerve in TNF-treated group at 1 week (Fig. 3a). Treatment with 200 pmol NR plus TNF completely prevented this increase of p62 (Fig. 3a). In addition, 200 pmol NR alone administration significantly decreased p62 protein level compared with the control group (Fig. 3b).

### Effects of TNF and NR on SIRT1 Protein Levels in Optic Nerve

**Fig. 4:**
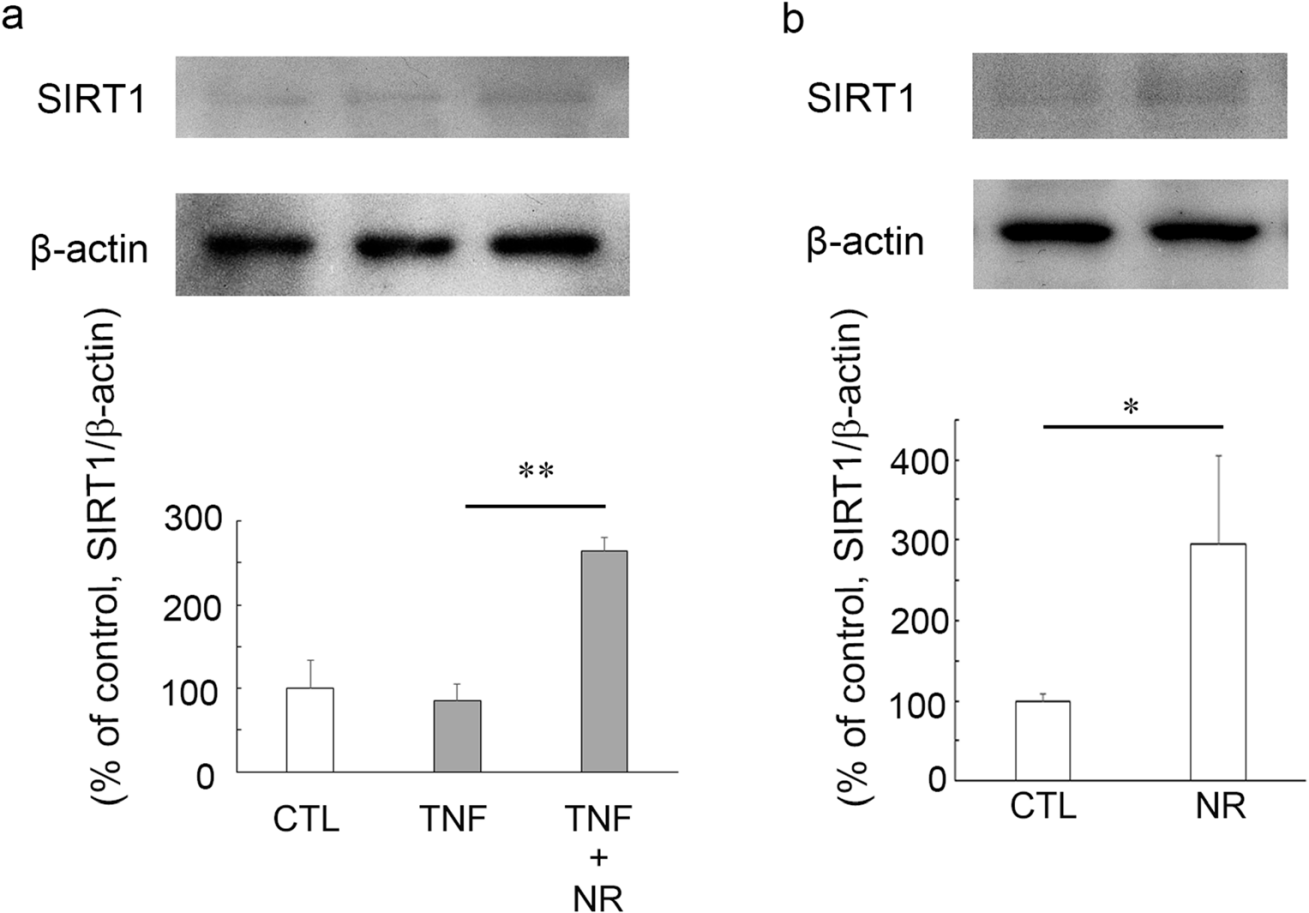
Immunoblotting in samples from optic nerves 1 week after injection. **a** Effects of TNF or 200 pmol NR + TNF on SIRT1 protein level. (CTL: *n* = 4, TNF: *n* = 4, TNF + NR: *n* = 4) (P** < 0.005). **b** Effects of 200 pmol NR on SIRT1 protein level. (CTL: *n* = 3, NR: *n* = 3) (P* < 0.05)

As we recently found [28], no significant change in SIRT1 protein level was seen in between TNF-treated group and PBS-treated group (Fig. 4a). However, treatment with 200 pmol NR plus TNF significantly increased the SIRT1 levels compared with TNF alone treatment (Fig. 4a). Moreover, 200 pmol NR alone administration significantly upregulated the SIRT1 levels compared with the control group (Fig. 4b).

### NRK1 in Retina and Optic Nerve

**Fig. 5:**
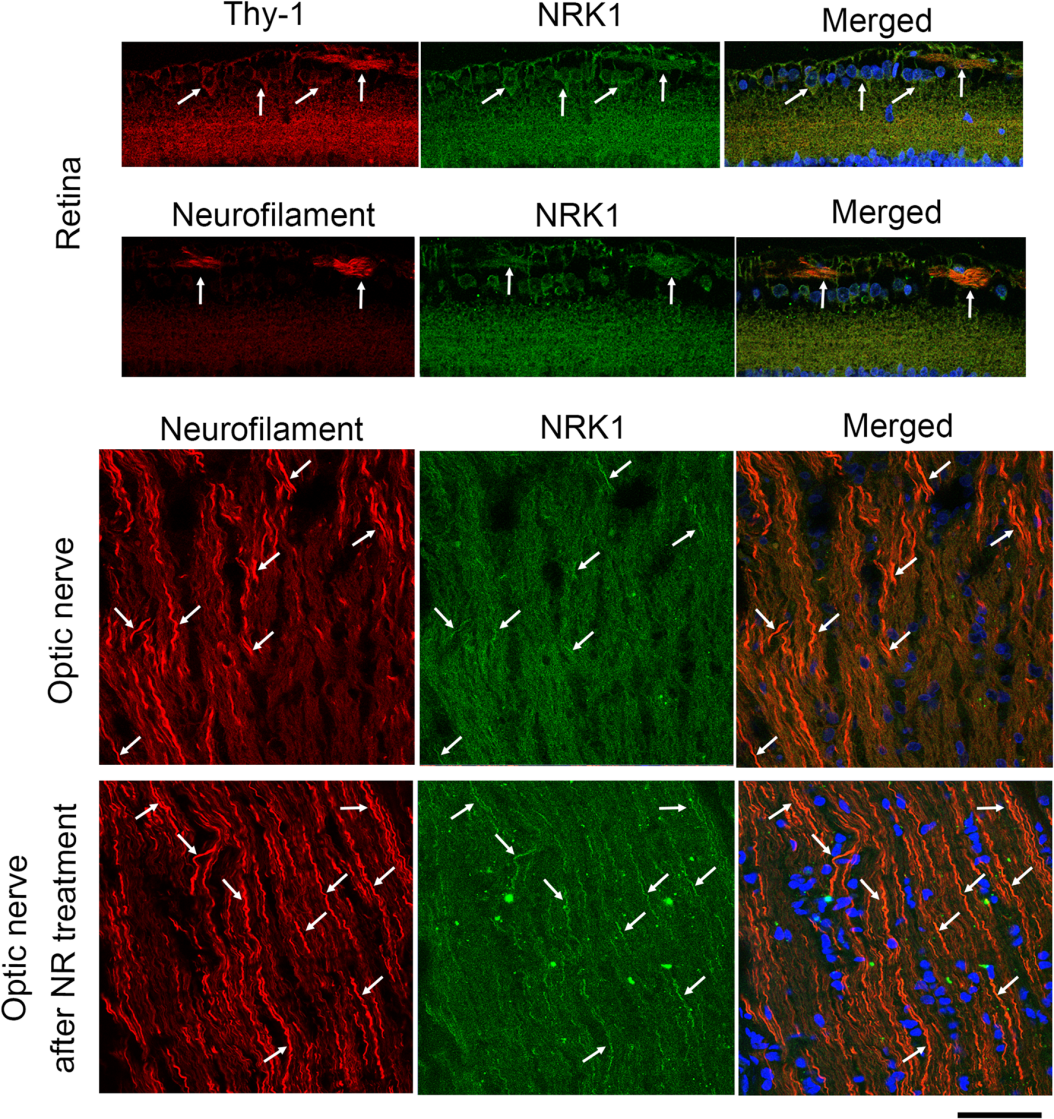
Immunohistochemistry in retina and optic nerve. NRK1-positive cells were colocalized with Thy-1-positive cells in normal retina. NRK1 immunoreactivity was colocalized with neurofilament immunoreactivity in normal retina. A few NRK1 immunoreactivities were colocalized with neurofilament immunoreactivity in normal optic nerve, but a lot of NRK immunopositive fibers were colocalized with neurofilament immunoreactivity in the NR-treated optic nerve. Arrows indicate colocalization. Scale bar = 50 μm

To examine the effect of NR and its metabolic pathway further, we investigated the localization of NRK1 in the retina and optic nerve. In the normal retina, the NRK1 immunoreactive pattern was similar to that of Thy-1 immunoreactivity (Fig. 5, upper panels). Most NRK1-positive cells were colocalized with Thy-1-positive cells (Fig. 5, upper panels). The NRK1 immunoreactivity was also observed in the nerve fiber layer, and these were colocalized with neurofilament immunoreactivity (Fig. 5, upper middle panel). In the optic nerve, the immunoreactivity of NRK1 was modest, but some immunopositive fibers were colocalized with neurofilament immunoreactivity (Fig. 5, lower middle panel). In the optic nerve after NR treatment, a lot of NRK1 immunopositive fibers were apparently colocalized with neurofilament immunoreactivity (Fig. 5, lower panel). In the retina after NR treatment, similar findings to the normal eyes were observed (Suppl. Fig. 1).

### Effects of TNF and NR on NRK1 Protein Levels in Optic Nerve

**Fig. 6:**
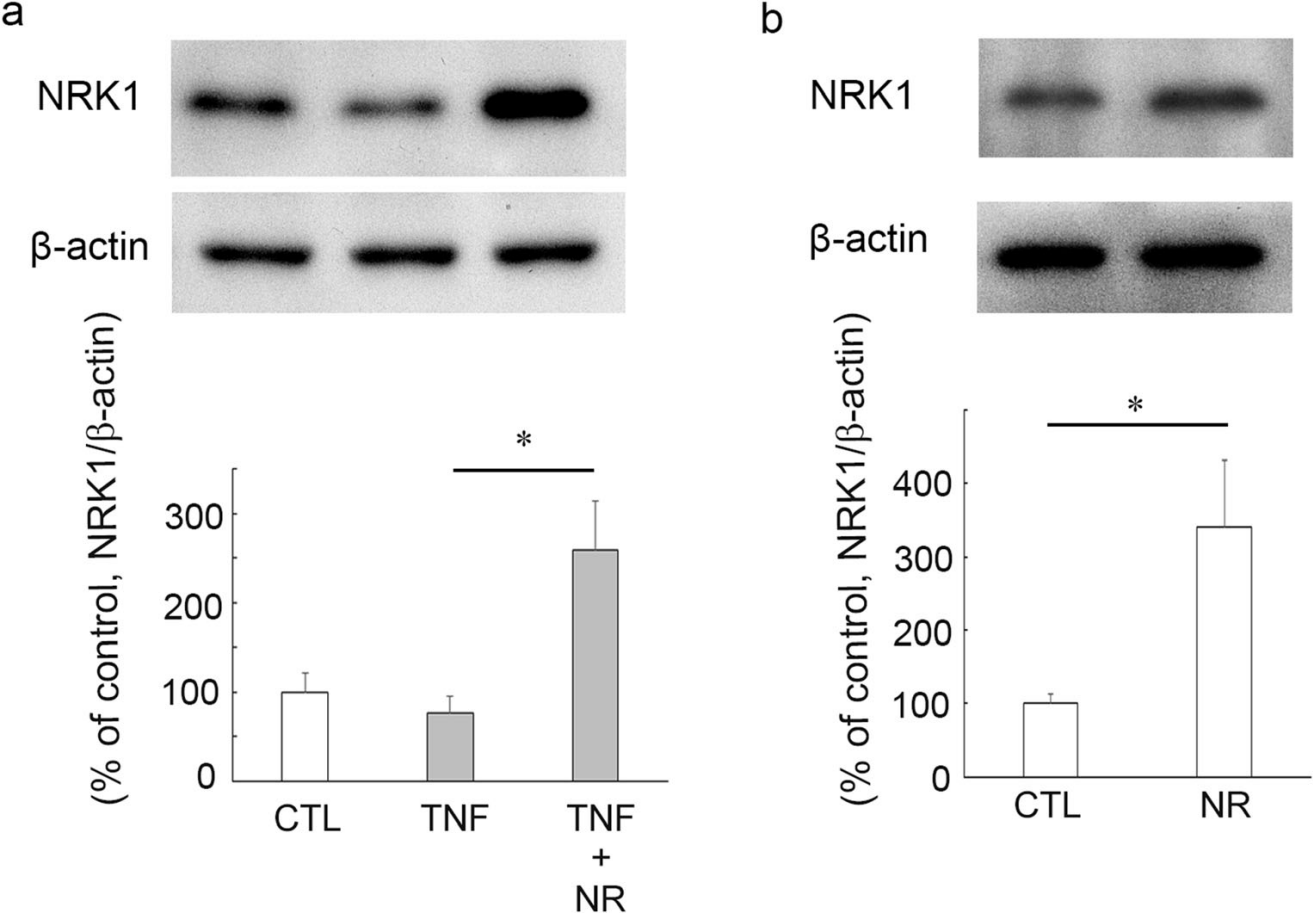
Immunoblotting in samples from optic nerves 1 week after injection. **a** Effects of TNF or 200 pmol NR + TNF on NRK1 protein level. (CTL: *n* = 4, TNF: *n* = 4, TNF + NR: *n* = 4) (P* < 0.05). **b** Effects of 200 pmol NR on NRK1 protein level. (CTL: *n* = 3, NR: *n* = 3) (P* < 0.05)

We next investigated the change in NRK1 expression in optic nerve. There was a tendency of decrease in NRK1 protein levels after TNF injection (Fig. 6a). Unexpectedly, treatment with 200 pmol NR plus TNF significantly increased the NRK1 levels compared with TNF alone treatment (Fig. 6a). Furthermore, 200 pmol NR alone administration significantly upregulated the NRK1 levels compared with the control group (Fig. 6b).

### Effects of an SIRT1 Inhibitor on p62 Protein Level in the NR plus TNF Treatment in Optic Nerve

**Fig. 7:**
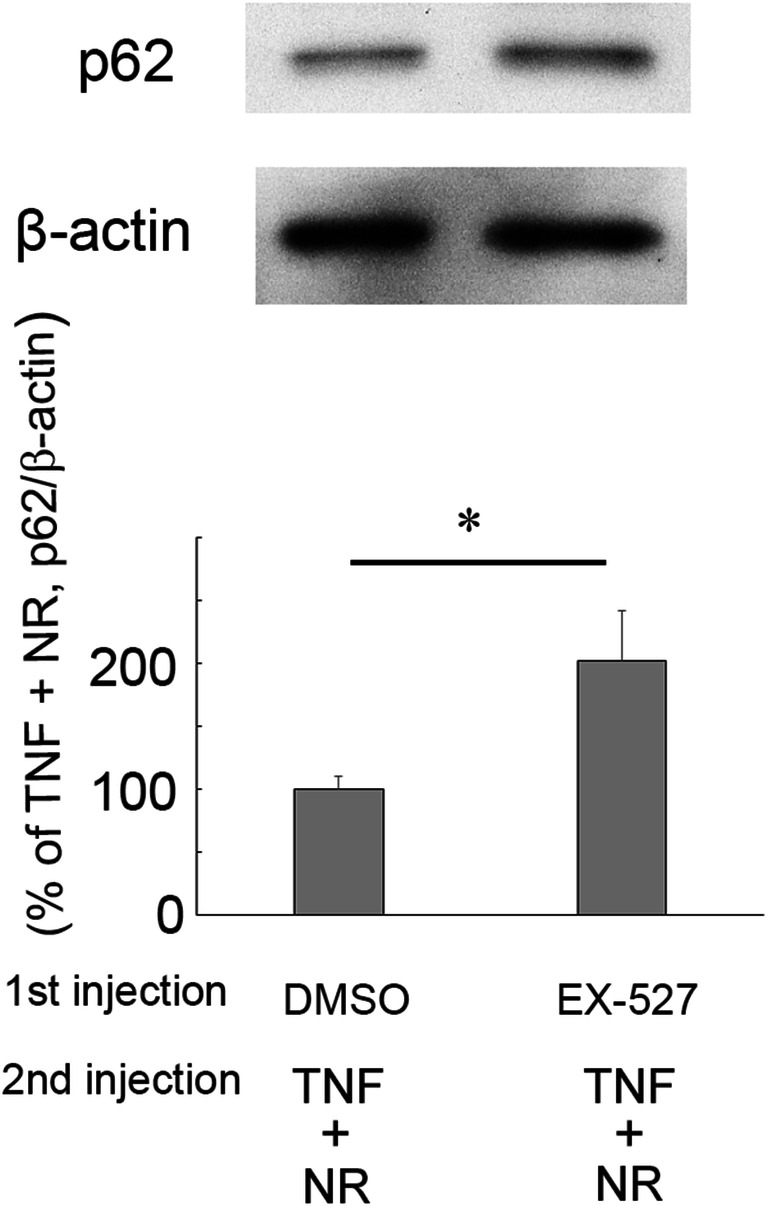
Immunoblotting in samples from optic nerves 1 week after injection. Effects of EX-527 on p62 protein level in the 200 pmol NR plus TNF-treated group. (pre-injection of DMSO and TNF + NR: *n* = 4, pre-injection of EX-527 and TNF + NR: *n* = 4) (P* < 0.05)

To investigate whether an inhibitor of SIRT1 alters autophagy status, pre-injection of EX-527 was performed before co-injection with 200 pmol NR and TNF. Pre-injection of EX-527, an inhibitor of SIRT1, significantly upregulated p62 levels as compared with NR plus TNF treatment group (Fig. 7).

## Discussion

The present study revealed that intravitreal injection of NR exerted substantial axonal protection in TNF-induced optic nerve degeneration. Recent studies have demonstrated beneficial effects of NR on central nervous system. For example, NR treatment prevented dopaminergic neuronal loss in Parkinson’s disease model flies [29]. Moreover, NR treatment ameliorated selective cognitive impairment in aged mice and decreased the number of amyloid beta plaques in cortex of Alzheimer’s disease model mice [30]. Furthermore, NR treatment decreased glial activation and delayed motor neuron loss in the spinal code of amyotrophic lateral sclerosis model mice [31]. In axons, it was previously reported that NR significantly delayed axonal degeneration in dorsal root ganglia neurons [5]. More recently, it was shown that NR prevented axonal degeneration induced by excitotoxicity in cortical neurons [32]. Collectively, NR may have a protective effect on several types of axons against distinct injury models.

Our previous study suggested that upregulated p62 levels indicate impairment of autophagic flux in optic nerve [8, 18, 27]. In the present study, upregulated p62 levels induced by TNF were significantly prevented by NR. NR significantly increased LC3-II protein levels in both the TNF-treated group and the control group. NR also significantly reduced p62 protein level compared with the basal level, implicating that NR can enhance autophagic flux. It is worthy to note that nicotinamide protects against palmitate-induced hepatotoxicity through SIRT1-dependent autophagy induction [33]. Since SIRT1 activators stimulate the autophagy [34, 35] and increased NAD^+^ stimulates SIRT1 activity [36], we speculated that this pathway may exist between NR and autophagy induction. Therefore, we further examined SIRT1 expression and found that NR upregulated SIRT1 protein level in optic nerve. Consistently, a very recent study demonstrated that treatment of NR upregulated SIRT1 activity and decreased neuroinflammation in the brains of Gulf War Illness mice [37], suggesting that NR can activate SIRT1 in certain neuronal system as well as in optic nerve. Moreover, our recent study demonstrated that an SIRT1 activator exerted axonal protection with upregulated autophagic status [28]. Furthermore, the current study found that the SIRT1 inhibitor significantly upregulated p62 level in the NR plus TNF treated group, implicating that inhibition of SIRT1 leads to autophagy impairment. Taken together, these findings suggest that NR attenuated axonal degeneration via SIRT1-autophagy pathway. In line with this concept, a previous study demonstrated the neuroprotective activity of cilostazol via SIRT1-autophagy activation in rat Parkinson’s disease model [38].

Although Nmnats were found in optic nerve [7–9], NRK1 has not been examined in retina and optic nerve. Thus, the present study firstly showed that NRK1 exists in RGCs and optic nerve axons. This finding makes it possible that locally applied exogenous NR can accelerate NAD biosynthesis and activate downstream effectors. Surprisingly, NR administration clearly boosted NRK1 levels in the TNF-treated eyes as well as the control eyes. The mechanism of this regulation is unclear, while the regulation of NRK2 in muscle has been proposed in response to various conditions [39]. One hypothesis posits that exogenous NR may recruit more NRK1, thereby accelerating conversion to downstream effectors.

In conclusion, NR exerts axonal protection against TNF-induced optic nerve degeneration with the possible upregulated NRK1 and through SIRT1-autophagy pathway.

## Electronic supplementary material

Supplemental figure 1 Immunohistochemistry in the NR-treated retina. NRK1-positive cells were colocalized with Thy-1-positive cells. NRK1 immunoreactivity was colocalized with neurofilament immunoreactivity. Arrows indicate colocalization. Scale bar = 50 μm. (PNG 980 kb).

High resolution image (TIF 4757 kb).

## Data Availability

All data generated or analyzed during this study are included in this published article.

## References

[1] Bogan KL, Brenner C (2008). Nicotinic acid, nicotinamide, and nicotinamide riboside: a molecular evaluation of NAD+ precursor vitamins in human nutrition. Annu Rev Nutr.

[2] Araki T, Sasaki Y, Milbrandt J (2004). Increased nuclear NAD biosynthesis and SIRT1 activation prevent axonal degeneration. Science.

[3] Wang J, Zhai Q, Chen Y, Lin E, Gu W, McBurney MW, He Z (2005). A local mechanism mediates NAD-dependent protection of axon degeneration. J Cell Biol.

[4] Gilley J, Coleman MP (2010). Endogenous Nmnat2 is an essential survival factor for maintenance of healthy axons. PLoS Biol.

[5] Sasaki Y, Araki T, Milbrandt J (2006). Stimulation of nicotinamide adenine dinucleotide biosynthetic pathways delays axonal degeneration after axotomy. J Neurosci.

[6] Press C, Milbrandt J (2008). Nmnat delays axonal degeneration caused by mitochondrial and oxidative stress. J Neurosci.

[7] Kitaoka Y, Hayashi Y, Kumai T, Takeda H, Munemasa Y, Fujino H, Kitaoka Y, Ueno S, Sadun AA, Lam TT (2009). Axonal and cell body protection by nicotinamide adenine dinucleotide in tumor necrosis factor-induced optic neuropathy. J Neuropathol Exp Neurol.

[8] Kitaoka Y, Munemasa Y, Kojima K, Hirano A, Ueno S, Takagi H (2013). Axonal protection by Nmnat3 overexpression with involvement of autophagy in optic nerve degeneration. Cell Death Dis.

[9] Gilley J, Adalbert R, Yu G, Coleman MP (2013). Rescue of peripheral and CNS axon defects in mice lacking NMNAT2. J Neurosci.

[10] Zhu Y, Zhang L, Sasaki Y, Milbrandt J, Gidday JM (2013). Protection of mouse retinal ganglion cell axons and soma from glaucomatous and ischemic injury by cytoplasmic overexpression of Nmnat1. Invest Ophthalmol Vis Sci.

[11] Williams PA, Harder JM, Foxworth NE, Cochran KE, Philip VM, Porciatti V, Smithies O, John SW (2017). Vitamin B3 modulates mitochondrial vulnerability and prevents glaucoma in aged mice. Science.

[12] Kouassi Nzoughet J, Chao de la Barca JM, Guehlouz K, Leruez S, Coulbault L, Allouche S, Bocca C, Muller J, Amati-Bonneau P, Gohier P, Bonneau D, Simard G, Milea D, Lenaers G, Procaccio V, Reynier P (2019). Nicotinamide deficiency in primary open-angle glaucoma. Invest Ophthalmol Vis Sci.

[13] Trammell SA, Schmidt MS, Weidemann BJ, Redpath P, Jaksch F, Dellinger RW, Li Z, Abel ED, Migaud ME, Brenner C (2016). Nicotinamide riboside is uniquely and orally bioavailable in mice and humans. Nat Commun.

[14] Ratajczak J, Joffraud M, Trammell SA, Ras R, Canela N, Boutant M, Kulkarni SS, Rodrigues M, Redpath P, Migaud ME, Auwerx J, Yanes O, Brenner C, Cantó C (2016). NRK1 controls nicotinamide mononucleotide and nicotinamide riboside metabolism in mammalian cells. Nat Commun.

[15] Zhu Y, Zhao KK, Tong Y, Zhou YL, Wang YX, Zhao PQ, Wang ZY (2016). Exogenous NAD(+) decreases oxidative stress and protects H_2_O_2_-treated RPE cells against necrotic death through the up-regulation of autophagy. Sci Rep.

[16] Sedlackova L, Korolchuk VI (2020). The crosstalk of NAD, ROS and autophagy in cellular health and ageing. Biogerontology.

[17] Li W, Zhu L, Ruan ZB, Wang MX, Ren Y, Lu W (2019). Nicotinamide protects chronic hypoxic myocardial cells through regulating mTOR pathway and inducing autophagy. Eur Rev Med Pharmacol Sci.

[18] Munemasa Y, Kitaoka Y (2015). Autophagy in axonal degeneration in glaucomatous optic neuropathy. Prog Retin Eye Res.

[19] Koch JC, Lingor P (2016). The role of autophagy in axonal degeneration of the optic nerve. Exp Eye Res.

[20] Russo R, Nucci C, Corasaniti MT, Bagetta G, Morrone LA (2015). Autophagy dysregulation and the fate of retinal ganglion cells in glaucomatous optic neuropathy. Prog Brain Res.

[21] Adornetto A, Parisi V, Morrone LA, Corasaniti MT, Bagetta G, Tonin P, Russo R (2020). The role of autophagy in glaucomatous optic neuropathy. Front Cell Dev Biol.

[22] Klionsky DJ, Abdelmohsen K, Abe A, Abedin MJ, Abeliovich H, Acevedo Arozena A, Adachi H, Adams CM, Adams PD, Adeli K, Adhihetty PJ, Adler SG, Agam G, Agarwal R, Aghi MK, Agnello M, Agostinis P, Aguilar PV, Aguirre-Ghiso J, Airoldi EM, Ait-Si-Ali S, Akematsu T, Akporiaye ET, al-Rubeai M, Albaiceta GM, Albanese C, Albani D, Albert ML, Aldudo J, Algül H, Alirezaei M, Alloza I, Almasan A, Almonte-Beceril M, Alnemri ES, Alonso C, Altan-Bonnet N, Altieri DC, Alvarez S, Alvarez-Erviti L, Alves S, Amadoro G, Amano A, Amantini C, Ambrosio S, Amelio I, Amer AO, Amessou M, Amon A, An Z, Anania FA, Andersen SU, Andley UP, Andreadi CK, Andrieu-Abadie N, Anel A, Ann DK, Anoopkumar-Dukie S, Antonioli M, Aoki H, Apostolova N, Aquila S, Aquilano K, Araki K, Arama E, Aranda A, Araya J, Arcaro A, Arias E, Arimoto H, Ariosa AR, Armstrong JL, Arnould T, Arsov I, Asanuma K, Askanas V, Asselin E, Atarashi R, Atherton SS, Atkin JD, Attardi LD, Auberger P, Auburger G, Aurelian L, Autelli R, Avagliano L, Avantaggiati ML, Avrahami L, Awale S, Azad N, Bachetti T, Backer JM, Bae DH, Bae JS, Bae ON, Bae SH, Baehrecke EH, Baek SH, Baghdiguian S, Bagniewska-Zadworna A, Bai H, Bai J, Bai XY, Bailly Y, Balaji KN, Balduini W, Ballabio A, Balzan R, Banerjee R, Bánhegyi G, Bao H, Barbeau B, Barrachina MD, Barreiro E, Bartel B, Bartolomé A, Bassham DC, Bassi MT, Bast RC, Basu A, Batista MT, Batoko H, Battino M, Bauckman K, Baumgarner BL, Bayer KU, Beale R, Beaulieu JF, Beck GR, Becker C, Beckham JD, Bédard PA, Bednarski PJ, Begley TJ, Behl C, Behrends C, Behrens GMN, Behrns KE, Bejarano E, Belaid A, Belleudi F, Bénard G, Berchem G, Bergamaschi D, Bergami M, Berkhout B, Berliocchi L, Bernard A, Bernard M, Bernassola F, Bertolotti A, Bess AS, Besteiro S, Bettuzzi S, Bhalla S, Bhattacharyya S, Bhutia SK, Biagosch C, Bianchi MW, Biard-Piechaczyk M, Billes V, Bincoletto C, Bingol B, Bird SW, Bitoun M, Bjedov I, Blackstone C, Blanc L, Blanco GA, Blomhoff HK, Boada-Romero E, Böckler S, Boes M, Boesze-Battaglia K, Boise LH, Bolino A, Boman A, Bonaldo P, Bordi M, Bosch J, Botana LM, Botti J, Bou G, Bouché M, Bouchecareilh M, Boucher MJ, Boulton ME, Bouret SG, Boya P, Boyer-Guittaut M, Bozhkov PV, Brady N, Braga VMM, Brancolini C, Braus GH, Bravo-San Pedro JM, Brennan LA, Bresnick EH, Brest P, Bridges D, Bringer MA, Brini M, Brito GC, Brodin B, Brookes PS, Brown EJ, Brown K, Broxmeyer HE, Bruhat A, Brum PC, Brumell JH, Brunetti-Pierri N, Bryson-Richardson RJ, Buch S, Buchan AM, Budak H, Bulavin DV, Bultman SJ, Bultynck G, Bumbasirevic V, Burelle Y, Burke RE, Burmeister M, Bütikofer P, Caberlotto L, Cadwell K, Cahova M, Cai D, Cai J, Cai Q, Calatayud S, Camougrand N, Campanella M, Campbell GR, Campbell M, Campello S, Candau R, Caniggia I, Cantoni L, Cao L, Caplan AB, Caraglia M, Cardinali C, Cardoso SM, Carew JS, Carleton LA, Carlin CR, Carloni S, Carlsson SR, Carmona-Gutierrez D, Carneiro LAM, Carnevali O, Carra S, Carrier A, Carroll B, Casas C, Casas J, Cassinelli G, Castets P, Castro-Obregon S, Cavallini G, Ceccherini I, Cecconi F, Cederbaum AI, Ceña V, Cenci S, Cerella C, Cervia D, Cetrullo S, Chaachouay H, Chae HJ, Chagin AS, Chai CY, Chakrabarti G, Chamilos G, Chan EYW, Chan MTV, Chandra D, Chandra P, Chang CP, Chang RCC, Chang TY, Chatham JC, Chatterjee S, Chauhan S, Che Y, Cheetham ME, Cheluvappa R, Chen CJ, Chen G, Chen GC, Chen G, Chen H, Chen JW, Chen JK, Chen M, Chen M, Chen P, Chen Q, Chen Q, Chen SD, Chen S, Chen SSL, Chen W, Chen WJ, Chen WQ, Chen W, Chen X, Chen YH, Chen YG, Chen Y, Chen Y, Chen Y, Chen YJ, Chen YQ, Chen Y, Chen Z, Chen Z, Cheng A, Cheng CHK, Cheng H, Cheong H, Cherry S, Chesney J, Cheung CHA, Chevet E, Chi HC, Chi SG, Chiacchiera F, Chiang HL, Chiarelli R, Chiariello M, Chieppa M, Chin LS, Chiong M, Chiu GNC, Cho DH, Cho SG, Cho WC, Cho YY, Cho YS, Choi AMK, Choi EJ, Choi EK, Choi J, Choi ME, Choi SI, Chou TF, Chouaib S, Choubey D, Choubey V, Chow KC, Chowdhury K, Chu CT, Chuang TH, Chun T, Chung H, Chung T, Chung YL, Chwae YJ, Cianfanelli V, Ciarcia R, Ciechomska IA, Ciriolo MR, Cirone M, Claerhout S, Clague MJ, Clària J, Clarke PGH, Clarke R, Clementi E, Cleyrat C, Cnop M, Coccia EM, Cocco T, Codogno P, Coers J, Cohen EEW, Colecchia D, Coletto L, Coll NS, Colucci-Guyon E, Comincini S, Condello M, Cook KL, Coombs GH, Cooper CD, Cooper JM, Coppens I, Corasaniti MT, Corazzari M, Corbalan R, Corcelle-Termeau E, Cordero MD, Corral-Ramos C, Corti O, Cossarizza A, Costelli P, Costes S, Cotman SL, Coto-Montes A, Cottet S, Couve E, Covey LR, Cowart LA, Cox JS, Coxon FP, Coyne CB, Cragg MS, Craven RJ, Crepaldi T, Crespo JL, Criollo A, Crippa V, Cruz MT, Cuervo AM, Cuezva JM, Cui T, Cutillas PR, Czaja MJ, Czyzyk-Krzeska MF, Dagda RK, Dahmen U, Dai C, Dai W, Dai Y, Dalby KN, Dalla Valle L, Dalmasso G, D'Amelio M, Damme M, Darfeuille-Michaud A, Dargemont C, Darley-Usmar VM, Dasarathy S, Dasgupta B, Dash S, Dass CR, Davey HM, Davids LM, Dávila D, Davis RJ, Dawson TM, Dawson VL, Daza P, de Belleroche J, de Figueiredo P, de Figueiredo RCBQ, de la Fuente J, de Martino L, de Matteis A, de Meyer GRY, de Milito A, de Santi M, de Souza W, de Tata V, de Zio D, Debnath J, Dechant R, Decuypere JP, Deegan S, Dehay B, del Bello B, del Re DP, Delage-Mourroux R, Delbridge LMD, Deldicque L, Delorme-Axford E, Deng Y, Dengjel J, Denizot M, Dent P, der CJ, Deretic V, Derrien B, Deutsch E, Devarenne TP, Devenish RJ, di Bartolomeo S, di Daniele N, di Domenico F, di Nardo A, di Paola S, di Pietro A, di Renzo L, DiAntonio A, Díaz-Araya G, Díaz-Laviada I, Diaz-Meco MT, Diaz-Nido J, Dickey CA, Dickson RC, Diederich M, Digard P, Dikic I, Dinesh-Kumar SP, Ding C, Ding WX, Ding Z, Dini L, Distler JHW, Diwan A, Djavaheri-Mergny M, Dmytruk K, Dobson RCJ, Doetsch V, Dokladny K, Dokudovskaya S, Donadelli M, Dong XC, Dong X, Dong Z, Donohue TM, Doran KS, D'Orazi G, Dorn GW, Dosenko V, Dridi S, Drucker L, du J, du LL, du L, du Toit A, Dua P, Duan L, Duann P, Dubey VK, Duchen MR, Duchosal MA, Duez H, Dugail I, Dumit VI, Duncan MC, Dunlop EA, Dunn WA, Dupont N, Dupuis L, Durán RV, Durcan TM, Duvezin-Caubet S, Duvvuri U, Eapen V, Ebrahimi-Fakhari D, Echard A, Eckhart L, Edelstein CL, Edinger AL, Eichinger L, Eisenberg T, Eisenberg-Lerner A, Eissa NT, el-Deiry WS, el-Khoury V, Elazar Z, Eldar-Finkelman H, Elliott CJH, Emanuele E, Emmenegger U, Engedal N, Engelbrecht AM, Engelender S, Enserink JM, Erdmann R, Erenpreisa J, Eri R, Eriksen JL, Erman A, Escalante R, Eskelinen EL, Espert L, Esteban-Martínez L, Evans TJ, Fabri M, Fabrias G, Fabrizi C, Facchiano A, Færgeman NJ, Faggioni A, Fairlie WD, Fan C, Fan D, Fan J, Fang S, Fanto M, Fanzani A, Farkas T, Faure M, Favier FB, Fearnhead H, Federici M, Fei E, Felizardo TC, Feng H, Feng Y, Feng Y, Ferguson TA, Fernández ÁF, Fernandez-Barrena MG, Fernandez-Checa JC, Fernández-López A, Fernandez-Zapico ME, Feron O, Ferraro E, Ferreira-Halder CV, Fesus L, Feuer R, Fiesel FC, Filippi-Chiela EC, Filomeni G, Fimia GM, Fingert JH, Finkbeiner S, Finkel T, Fiorito F, Fisher PB, Flajolet M, Flamigni F, Florey O, Florio S, Floto RA, Folini M, Follo C, Fon EA, Fornai F, Fortunato F, Fraldi A, Franco R, Francois A, François A, Frankel LB, Fraser IDC, Frey N, Freyssenet DG, Frezza C, Friedman SL, Frigo DE, Fu D, Fuentes JM, Fueyo J, Fujitani Y, Fujiwara Y, Fujiya M, Fukuda M, Fulda S, Fusco C, Gabryel B, Gaestel M, Gailly P, Gajewska M, Galadari S, Galili G, Galindo I, Galindo MF, Galliciotti G, Galluzzi L, Galluzzi L, Galy V, Gammoh N, Gandy S, Ganesan AK, Ganesan S, Ganley IG, Gannagé M, Gao FB, Gao F, Gao JX, García Nannig L, García Véscovi E, Garcia-Macía M, Garcia-Ruiz C, Garg AD, Garg PK, Gargini R, Gassen NC, Gatica D, Gatti E, Gavard J, Gavathiotis E, Ge L, Ge P, Ge S, Gean PW, Gelmetti V, Genazzani AA, Geng J, Genschik P, Gerner L, Gestwicki JE, Gewirtz DA, Ghavami S, Ghigo E, Ghosh D, Giammarioli AM, Giampieri F, Giampietri C, Giatromanolaki A, Gibbings DJ, Gibellini L, Gibson SB, Ginet V, Giordano A, Giorgini F, Giovannetti E, Girardin SE, Gispert S, Giuliano S, Gladson CL, Glavic A, Gleave M, Godefroy N, Gogal RM, Gokulan K, Goldman GH, Goletti D, Goligorsky MS, Gomes AV, Gomes LC, Gomez H, Gomez-Manzano C, Gómez-Sánchez R, Gonçalves DAP, Goncu E, Gong Q, Gongora C, Gonzalez CB, Gonzalez-Alegre P, Gonzalez-Cabo P, González-Polo RA, Goping IS, Gorbea C, Gorbunov NV, Goring DR, Gorman AM, Gorski SM, Goruppi S, Goto-Yamada S, Gotor C, Gottlieb RA, Gozes I, Gozuacik D, Graba Y, Graef M, Granato GE, Grant GD, Grant S, Gravina GL, Green DR, Greenhough A, Greenwood MT, Grimaldi B, Gros F, Grose C, Groulx JF, Gruber F, Grumati P, Grune T, Guan JL, Guan KL, Guerra B, Guillen C, Gulshan K, Gunst J, Guo C, Guo L, Guo M, Guo W, Guo XG, Gust AA, Gustafsson ÅB, Gutierrez E, Gutierrez MG, Gwak HS, Haas A, Haber JE, Hadano S, Hagedorn M, Hahn DR, Halayko AJ, Hamacher-Brady A, Hamada K, Hamai A, Hamann A, Hamasaki M, Hamer I, Hamid Q, Hammond EM, Han F, Han W, Handa JT, Hanover JA, Hansen M, Harada M, Harhaji-Trajkovic L, Harper JW, Harrath AH, Harris AL, Harris J, Hasler U, Hasselblatt P, Hasui K, Hawley RG, Hawley TS, He C, He CY, He F, He G, He RR, He XH, He YW, He YY, Heath JK, Hébert MJ, Heinzen RA, Helgason GV, Hensel M, Henske EP, Her C, Herman PK, Hernández A, Hernandez C, Hernández-Tiedra S, Hetz C, Hiesinger PR, Higaki K, Hilfiker S, Hill BG, Hill JA, Hill WD, Hino K, Hofius D, Hofman P, Höglinger GU, Höhfeld J, Holz MK, Hong Y, Hood DA, Hoozemans JJM, Hoppe T, Hsu C, Hsu CY, Hsu LC, Hu D, Hu G, Hu HM, Hu H, Hu MC, Hu YC, Hu ZW, Hua F, Hua Y, Huang C, Huang HL, Huang KH, Huang KY, Huang S, Huang S, Huang WP, Huang YR, Huang Y, Huang Y, Huber TB, Huebbe P, Huh WK, Hulmi JJ, Hur GM, Hurley JH, Husak Z, Hussain SNA, Hussain S, Hwang JJ, Hwang S, Hwang TIS, Ichihara A, Imai Y, Imbriano C, Inomata M, Into T, Iovane V, Iovanna JL, Iozzo RV, Ip NY, Irazoqui JE, Iribarren P, Isaka Y, Isakovic AJ, Ischiropoulos H, Isenberg JS, Ishaq M, Ishida H, Ishii I, Ishmael JE, Isidoro C, Isobe KI, Isono E, Issazadeh-Navikas S, Itahana K, Itakura E, Ivanov AI, Iyer AKV, Izquierdo JM, Izumi Y, Izzo V, Jäättelä M, Jaber N, Jackson DJ, Jackson WT, Jacob TG, Jacques TS, Jagannath C, Jain A, Jana NR, Jang BK, Jani A, Janji B, Jannig PR, Jansson PJ, Jean S, Jendrach M, Jeon JH, Jessen N, Jeung EB, Jia K, Jia L, Jiang H, Jiang H, Jiang L, Jiang T, Jiang X, Jiang X, Jiang X, Jiang Y, Jiang Y, Jiménez A, Jin C, Jin H, Jin L, Jin M, Jin S, Jinwal UK, Jo EK, Johansen T, Johnson DE, Johnson GVW, Johnson JD, Jonasch E, Jones C, Joosten LAB, Jordan J, Joseph AM, Joseph B, Joubert AM, Ju D, Ju J, Juan HF, Juenemann K, Juhász G, Jung HS, Jung JU, Jung YK, Jungbluth H, Justice MJ, Jutten B, Kaakoush NO, Kaarniranta K, Kaasik A, Kabuta T, Kaeffer B, Kågedal K, Kahana A, Kajimura S, Kakhlon O, Kalia M, Kalvakolanu DV, Kamada Y, Kambas K, Kaminskyy VO, Kampinga HH, Kandouz M, Kang C, Kang R, Kang TC, Kanki T, Kanneganti TD, Kanno H, Kanthasamy AG, Kantorow M, Kaparakis-Liaskos M, Kapuy O, Karantza V, Karim MR, Karmakar P, Kaser A, Kaushik S, Kawula T, Kaynar AM, Ke PY, Ke ZJ, Kehrl JH, Keller KE, Kemper JK, Kenworthy AK, Kepp O, Kern A, Kesari S, Kessel D, Ketteler R, Kettelhut IC, Khambu B, Khan MM, Khandelwal VKM, Khare S, Kiang JG, Kiger AA, Kihara A, Kim AL, Kim CH, Kim DR, Kim DH, Kim EK, Kim HY, Kim HR, Kim JS, Kim JH, Kim JC, Kim JH, Kim KW, Kim MD, Kim MM, Kim PK, Kim SW, Kim SY, Kim YS, Kim Y, Kimchi A, Kimmelman AC, Kimura T, King JS, Kirkegaard K, Kirkin V, Kirshenbaum LA, Kishi S, Kitajima Y, Kitamoto K, Kitaoka Y, Kitazato K, Kley RA, Klimecki WT, Klinkenberg M, Klucken J, Knævelsrud H, Knecht E, Knuppertz L, Ko JL, Kobayashi S, Koch JC, Koechlin-Ramonatxo C, Koenig U, Koh YH, Köhler K, Kohlwein SD, Koike M, Komatsu M, Kominami E, Kong D, Kong HJ, Konstantakou EG, Kopp BT, Korcsmaros T, Korhonen L, Korolchuk VI, Koshkina NV, Kou Y, Koukourakis MI, Koumenis C, Kovács AL, Kovács T, Kovacs WJ, Koya D, Kraft C, Krainc D, Kramer H, Kravic-Stevovic T, Krek W, Kretz-Remy C, Krick R, Krishnamurthy M, Kriston-Vizi J, Kroemer G, Kruer MC, Kruger R, Ktistakis NT, Kuchitsu K, Kuhn C, Kumar AP, Kumar A, Kumar A, Kumar D, Kumar D, Kumar R, Kumar S, Kundu M, Kung HJ, Kuno A, Kuo SH, Kuret J, Kurz T, Kwok T, Kwon TK, Kwon YT, Kyrmizi I, la Spada AR, Lafont F, Lahm T, Lakkaraju A, Lam T, Lamark T, Lancel S, Landowski TH, Lane DJR, Lane JD, Lanzi C, Lapaquette P, Lapierre LR, Laporte J, Laukkarinen J, Laurie GW, Lavandero S, Lavie L, LaVoie MJ, Law BYK, Law HKW, Law KB, Layfield R, Lazo PA, le Cam L, le Roch KG, le Stunff H, Leardkamolkarn V, Lecuit M, Lee BH, Lee CH, Lee EF, Lee GM, Lee HJ, Lee H, Lee JK, Lee J, Lee JH, Lee JH, Lee M, Lee MS, Lee PJ, Lee SW, Lee SJ, Lee SJ, Lee SY, Lee SH, Lee SS, Lee SJ, Lee S, Lee YR, Lee YJ, Lee YH, Leeuwenburgh C, Lefort S, Legouis R, Lei J, Lei QY, Leib DA, Leibowitz G, Lekli I, Lemaire SD, Lemasters JJ, Lemberg MK, Lemoine A, Leng S, Lenz G, Lenzi P, Lerman LO, Lettieri Barbato D, Leu JIJ, Leung HY, Levine B, Lewis PA, Lezoualc'h F, Li C, Li F, Li FJ, Li J, Li K, Li L, Li M, Li M, Li Q, Li R, Li S, Li W, Li W, Li X, Li Y, Lian J, Liang C, Liang Q, Liao Y, Liberal J, Liberski PP, Lie P, Lieberman AP, Lim HJ, Lim KL, Lim K, Lima RT, Lin CS, Lin CF, Lin F, Lin F, Lin FC, Lin K, Lin KH, Lin PH, Lin T, Lin WW, Lin YS, Lin Y, Linden R, Lindholm D, Lindqvist LM, Lingor P, Linkermann A, Liotta LA, Lipinski MM, Lira VA, Lisanti MP, Liton PB, Liu B, Liu C, Liu CF, Liu F, Liu HJ, Liu J, Liu JJ, Liu JL, Liu K, Liu L, Liu L, Liu Q, Liu RY, Liu S, Liu S, Liu W, Liu XD, Liu X, Liu XH, Liu X, Liu X, Liu X, Liu Y, Liu Y, Liu Z, Liu Z, Liuzzi JP, Lizard G, Ljujic M, Lodhi IJ, Logue SE, Lokeshwar BL, Long YC, Lonial S, Loos B, López-Otín C, López-Vicario C, Lorente M, Lorenzi PL, Lõrincz P, Los M, Lotze MT, Lovat PE, Lu B, Lu B, Lu J, Lu Q, Lu SM, Lu S, Lu Y, Luciano F, Luckhart S, Lucocq JM, Ludovico P, Lugea A, Lukacs NW, Lum JJ, Lund AH, Luo H, Luo J, Luo S, Luparello C, Lyons T, Ma J, Ma Y, Ma Y, Ma Z, Machado J, Machado-Santelli GM, Macian F, MacIntosh GC, MacKeigan JP, Macleod KF, MacMicking JD, MacMillan-Crow LA, Madeo F, Madesh M, Madrigal-Matute J, Maeda A, Maeda T, Maegawa G, Maellaro E, Maes H, Magariños M, Maiese K, Maiti TK, Maiuri L, Maiuri MC, Maki CG, Malli R, Malorni W, Maloyan A, Mami-Chouaib F, Man N, Mancias JD, Mandelkow EM, Mandell MA, Manfredi AA, Manié SN, Manzoni C, Mao K, Mao Z, Mao ZW, Marambaud P, Marconi AM, Marelja Z, Marfe G, Margeta M, Margittai E, Mari M, Mariani FV, Marin C, Marinelli S, Mariño G, Markovic I, Marquez R, Martelli AM, Martens S, Martin KR, Martin SJ, Martin S, Martin-Acebes MA, Martín-Sanz P, Martinand-Mari C, Martinet W, Martinez J, Martinez-Lopez N, Martinez-Outschoorn U, Martínez-Velázquez M, Martinez-Vicente M, Martins WK, Mashima H, Mastrianni JA, Matarese G, Matarrese P, Mateo R, Matoba S, Matsumoto N, Matsushita T, Matsuura A, Matsuzawa T, Mattson MP, Matus S, Maugeri N, Mauvezin C, Mayer A, Maysinger D, Mazzolini GD, McBrayer MK, McCall K, McCormick C, McInerney GM, McIver SC, McKenna S, McMahon JJ, McNeish IA, Mechta-Grigoriou F, Medema JP, Medina DL, Megyeri K, Mehrpour M, Mehta JL, Mei Y, Meier UC, Meijer AJ, Meléndez A, Melino G, Melino S, de Melo EJT, Mena MA, Meneghini MD, Menendez JA, Menezes R, Meng L, Meng LH, Meng S, Menghini R, Menko AS, Menna-Barreto RFS, Menon MB, Meraz-Ríos MA, Merla G, Merlini L, Merlot AM, Meryk A, Meschini S, Meyer JN, Mi MT, Miao CY, Micale L, Michaeli S, Michiels C, Migliaccio AR, Mihailidou AS, Mijaljica D, Mikoshiba K, Milan E, Miller-Fleming L, Mills GB, Mills IG, Minakaki G, Minassian BA, Ming XF, Minibayeva F, Minina EA, Mintern JD, Minucci S, Miranda-Vizuete A, Mitchell CH, Miyamoto S, Miyazawa K, Mizushima N, Mnich K, Mograbi B, Mohseni S, Moita LF, Molinari M, Molinari M, Møller AB, Mollereau B, Mollinedo F, Mongillo M, Monick MM, Montagnaro S, Montell C, Moore DJ, Moore MN, Mora-Rodriguez R, Moreira PI, Morel E, Morelli MB, Moreno S, Morgan MJ, Moris A, Moriyasu Y, Morrison JL, Morrison LA, Morselli E, Moscat J, Moseley PL, Mostowy S, Motori E, Mottet D, Mottram JC, Moussa CEH, Mpakou VE, Mukhtar H, Mulcahy Levy JM, Muller S, Muñoz-Moreno R, Muñoz-Pinedo C, Münz C, Murphy ME, Murray JT, Murthy A, Mysorekar IU, Nabi IR, Nabissi M, Nader GA, Nagahara Y, Nagai Y, Nagata K, Nagelkerke A, Nagy P, Naidu SR, Nair S, Nakano H, Nakatogawa H, Nanjundan M, Napolitano G, Naqvi NI, Nardacci R, Narendra DP, Narita M, Nascimbeni AC, Natarajan R, Navegantes LC, Nawrocki ST, Nazarko TY, Nazarko VY, Neill T, Neri LM, Netea MG, Netea-Maier RT, Neves BM, Ney PA, Nezis IP, Nguyen HTT, Nguyen HP, Nicot AS, Nilsen H, Nilsson P, Nishimura M, Nishino I, Niso-Santano M, Niu H, Nixon RA, Njar VCO, Noda T, Noegel AA, Nolte EM, Norberg E, Norga KK, Noureini SK, Notomi S, Notterpek L, Nowikovsky K, Nukina N, Nürnberger T, O'Donnell VB, O'Donovan T, O'Dwyer PJ, Oehme I, Oeste CL, Ogawa M, Ogretmen B, Ogura Y, Oh YJ, Ohmuraya M, Ohshima T, Ojha R, Okamoto K, Okazaki T, Oliver FJ, Ollinger K, Olsson S, Orban DP, Ordonez P, Orhon I, Orosz L, O'Rourke EJ, Orozco H, Ortega AL, Ortona E, Osellame LD, Oshima J, Oshima S, Osiewacz HD, Otomo T, Otsu K, Ou JHJ, Outeiro TF, Ouyang DY, Ouyang H, Overholtzer M, Ozbun MA, Ozdinler PH, Ozpolat B, Pacelli C, Paganetti P, Page G, Pages G, Pagnini U, Pajak B, Pak SC, Pakos-Zebrucka K, Pakpour N, Palková Z, Palladino F, Pallauf K, Pallet N, Palmieri M, Paludan SR, Palumbo C, Palumbo S, Pampliega O, Pan H, Pan W, Panaretakis T, Pandey A, Pantazopoulou A, Papackova Z, Papademetrio DL, Papassideri I, Papini A, Parajuli N, Pardo J, Parekh VV, Parenti G, Park JI, Park J, Park OK, Parker R, Parlato R, Parys JB, Parzych KR, Pasquet JM, Pasquier B, Pasumarthi KBS, Patschan D, Patterson C, Pattingre S, Pattison S, Pause A, Pavenstädt H, Pavone F, Pedrozo Z, Peña FJ, Peñalva MA, Pende M, Peng J, Penna F, Penninger JM, Pensalfini A, Pepe S, Pereira GJS, Pereira PC, Pérez-de la Cruz V, Pérez-Pérez ME, Pérez-Rodríguez D, Pérez-Sala D, Perier C, Perl A, Perlmutter DH, Perrotta I, Pervaiz S, Pesonen M, Pessin JE, Peters GJ, Petersen M, Petrache I, Petrof BJ, Petrovski G, Phang JM, Piacentini M, Pierdominici M, Pierre P, Pierrefite-Carle V, Pietrocola F, Pimentel-Muiños FX, Pinar M, Pineda B, Pinkas-Kramarski R, Pinti M, Pinton P, Piperdi B, Piret JM, Platanias LC, Platta HW, Plowey ED, Pöggeler S, Poirot M, Polčic P, Poletti A, Poon AH, Popelka H, Popova B, Poprawa I, Poulose SM, Poulton J, Powers SK, Powers T, Pozuelo-Rubio M, Prak K, Prange R, Prescott M, Priault M, Prince S, Proia RL, Proikas-Cezanne T, Prokisch H, Promponas VJ, Przyklenk K, Puertollano R, Pugazhenthi S, Puglielli L, Pujol A, Puyal J, Pyeon D, Qi X, Qian WB, Qin ZH, Qiu Y, Qu Z, Quadrilatero J, Quinn F, Raben N, Rabinowich H, Radogna F, Ragusa MJ, Rahmani M, Raina K, Ramanadham S, Ramesh R, Rami A, Randall-Demllo S, Randow F, Rao H, Rao VA, Rasmussen BB, Rasse TM, Ratovitski EA, Rautou PE, Ray SK, Razani B, Reed BH, Reggiori F, Rehm M, Reichert AS, Rein T, Reiner DJ, Reits E, Ren J, Ren X, Renna M, Reusch JEB, Revuelta JL, Reyes L, Rezaie AR, Richards RI, Richardson DR, Richetta C, Riehle MA, Rihn BH, Rikihisa Y, Riley BE, Rimbach G, Rippo MR, Ritis K, Rizzi F, Rizzo E, Roach PJ, Robbins J, Roberge M, Roca G, Roccheri MC, Rocha S, Rodrigues CMP, Rodríguez CI, de Cordoba SR, Rodriguez-Muela N, Roelofs J, Rogov VV, Rohn TT, Rohrer B, Romanelli D, Romani L, Romano PS, Roncero MIG, Rosa JL, Rosello A, Rosen KV, Rosenstiel P, Rost-Roszkowska M, Roth KA, Roué G, Rouis M, Rouschop KM, Ruan DT, Ruano D, Rubinsztein DC, Rucker EB, Rudich A, Rudolf E, Rudolf R, Ruegg MA, Ruiz-Roldan C, Ruparelia AA, Rusmini P, Russ DW, Russo GL, Russo G, Russo R, Rusten TE, Ryabovol V, Ryan KM, Ryter SW, Sabatini DM, Sacher M, Sachse C, Sack MN, Sadoshima J, Saftig P, Sagi-Eisenberg R, Sahni S, Saikumar P, Saito T, Saitoh T, Sakakura K, Sakoh-Nakatogawa M, Sakuraba Y, Salazar-Roa M, Salomoni P, Saluja AK, Salvaterra PM, Salvioli R, Samali A, Sanchez AMJ, Sánchez-Alcázar JA, Sanchez-Prieto R, Sandri M, Sanjuan MA, Santaguida S, Santambrogio L, Santoni G, dos Santos CN, Saran S, Sardiello M, Sargent G, Sarkar P, Sarkar S, Sarrias MR, Sarwal MM, Sasakawa C, Sasaki M, Sass M, Sato K, Sato M, Satriano J, Savaraj N, Saveljeva S, Schaefer L, Schaible UE, Scharl M, Schatzl HM, Schekman R, Scheper W, Schiavi A, Schipper HM, Schmeisser H, Schmidt J, Schmitz I, Schneider BE, Schneider EM, Schneider JL, Schon EA, Schönenberger MJ, Schönthal AH, Schorderet DF, Schröder B, Schuck S, Schulze RJ, Schwarten M, Schwarz TL, Sciarretta S, Scotto K, Scovassi AI, Screaton RA, Screen M, Seca H, Sedej S, Segatori L, Segev N, Seglen PO, Seguí-Simarro JM, Segura-Aguilar J, Seki E, Seiliez I, Sell C, Semenkovich CF, Semenza GL, Sen U, Serra AL, Serrano-Puebla A, Sesaki H, Setoguchi T, Settembre C, Shacka JJ, Shajahan-Haq AN, Shapiro IM, Sharma S, She H, Shen CKJ, Shen CC, Shen HM, Shen S, Shen W, Sheng R, Sheng X, Sheng ZH, Shepherd TG, Shi J, Shi Q, Shi Q, Shi Y, Shibutani S, Shibuya K, Shidoji Y, Shieh JJ, Shih CM, Shimada Y, Shimizu S, Shin DW, Shinohara ML, Shintani M, Shintani T, Shioi T, Shirabe K, Shiri-Sverdlov R, Shirihai O, Shore GC, Shu CW, Shukla D, Sibirny AA, Sica V, Sigurdson CJ, Sigurdsson EM, Sijwali PS, Sikorska B, Silveira WA, Silvente-Poirot S, Silverman GA, Simak J, Simmet T, Simon AK, Simon HU, Simone C, Simons M, Simonsen A, Singh R, Singh SV, Singh SK, Sinha D, Sinha S, Sinicrope FA, Sirko A, Sirohi K, Sishi BJN, Sittler A, Siu PM, Sivridis E, Skwarska A, Slack R, Slaninová I, Slavov N, Smaili SS, Smalley KSM, Smith DR, Soenen SJ, Soleimanpour SA, Solhaug A, Somasundaram K, Son JH, Sonawane A, Song C, Song F, Song HK, Song JX, Song W, Soo KY, Sood AK, Soong TW, Soontornniyomkij V, Sorice M, Sotgia F, Soto-Pantoja DR, Sotthibundhu A, Sousa MJ, Spaink HP, Span PN, Spang A, Sparks JD, Speck PG, Spector SA, Spies CD, Springer W, Clair DS, Stacchiotti A, Staels B, Stang MT, Starczynowski DT, Starokadomskyy P, Steegborn C, Steele JW, Stefanis L, Steffan J, Stellrecht CM, Stenmark H, Stepkowski TM, Stern ST, Stevens C, Stockwell BR, Stoka V, Storchova Z, Stork B, Stratoulias V, Stravopodis DJ, Strnad P, Strohecker AM, Ström AL, Stromhaug P, Stulik J, Su YX, Su Z, Subauste CS, Subramaniam S, Sue CM, Suh SW, Sui X, Sukseree S, Sulzer D, Sun FL, Sun J, Sun J, Sun SY, Sun Y, Sun Y, Sun Y, Sundaramoorthy V, Sung J, Suzuki H, Suzuki K, Suzuki N, Suzuki T, Suzuki YJ, Swanson MS, Swanton C, Swärd K, Swarup G, Sweeney ST, Sylvester PW, Szatmari Z, Szegezdi E, Szlosarek PW, Taegtmeyer H, Tafani M, Taillebourg E, Tait SWG, Takacs-Vellai K, Takahashi Y, Takáts S, Takemura G, Takigawa N, Talbot NJ, Tamagno E, Tamburini J, Tan CP, Tan L, Tan ML, Tan M, Tan YJ, Tanaka K, Tanaka M, Tang D, Tang D, Tang G, Tanida I, Tanji K, Tannous BA, Tapia JA, Tasset-Cuevas I, Tatar M, Tavassoly I, Tavernarakis N, Taylor A, Taylor GS, Taylor GA, Taylor JP, Taylor MJ, Tchetina EV, Tee AR, Teixeira-Clerc F, Telang S, Tencomnao T, Teng BB, Teng RJ, Terro F, Tettamanti G, Theiss AL, Theron AE, Thomas KJ, Thomé MP, Thomes PG, Thorburn A, Thorner J, Thum T, Thumm M, Thurston TLM, Tian L, Till A, Ting JPY, Titorenko VI, Toker L, Toldo S, Tooze SA, Topisirovic I, Torgersen ML, Torosantucci L, Torriglia A, Torrisi MR, Tournier C, Towns R, Trajkovic V, Travassos LH, Triola G, Tripathi DN, Trisciuoglio D, Troncoso R, Trougakos IP, Truttmann AC, Tsai KJ, Tschan MP, Tseng YH, Tsukuba T, Tsung A, Tsvetkov AS, Tu S, Tuan HY, Tucci M, Tumbarello DA, Turk B, Turk V, Turner RFB, Tveita AA, Tyagi SC, Ubukata M, Uchiyama Y, Udelnow A, Ueno T, Umekawa M, Umemiya-Shirafuji R, Underwood BR, Ungermann C, Ureshino RP, Ushioda R, Uversky VN, Uzcátegui NL, Vaccari T, Vaccaro MI, Váchová L, Vakifahmetoglu-Norberg H, Valdor R, Valente EM, Vallette F, Valverde AM, van den Berghe G, van den Bosch L, van den Brink GR, van der Goot FG, van der Klei IJ, van der Laan LJW, van Doorn WG, van Egmond M, van Golen KL, van Kaer L, van Lookeren Campagne M, Vandenabeele P, Vandenberghe W, Vanhorebeek I, Varela-Nieto I, Vasconcelos MH, Vasko R, Vavvas DG, Vega-Naredo I, Velasco G, Velentzas AD, Velentzas PD, Vellai T, Vellenga E, Vendelbo MH, Venkatachalam K, Ventura N, Ventura S, Veras PST, Verdier M, Vertessy BG, Viale A, Vidal M, Vieira HLA, Vierstra RD, Vigneswaran N, Vij N, Vila M, Villar M, Villar VH, Villarroya J, Vindis C, Viola G, Viscomi MT, Vitale G, Vogl DT, Voitsekhovskaja OV, von Haefen C, von Schwarzenberg K, Voth DE, Vouret-Craviari V, Vuori K, Vyas JM, Waeber C, Walker CL, Walker MJ, Walter J, Wan L, Wan X, Wang B, Wang C, Wang CY, Wang C, Wang C, Wang C, Wang D, Wang F, Wang F, Wang G, Wang HJ, Wang H, Wang HG, Wang H, Wang HD, Wang J, Wang J, Wang M, Wang MQ, Wang PY, Wang P, Wang RC, Wang S, Wang TF, Wang X, Wang XJ, Wang XW, Wang X, Wang X, Wang Y, Wang Y, Wang Y, Wang YJ, Wang Y, Wang Y, Wang YT, Wang Y, Wang ZN, Wappner P, Ward C, Ward DMV, Warnes G, Watada H, Watanabe Y, Watase K, Weaver TE, Weekes CD, Wei J, Weide T, Weihl CC, Weindl G, Weis SN, Wen L, Wen X, Wen Y, Westermann B, Weyand CM, White AR, White E, Whitton JL, Whitworth AJ, Wiels J, Wild F, Wildenberg ME, Wileman T, Wilkinson DS, Wilkinson S, Willbold D, Williams C, Williams K, Williamson PR, Winklhofer KF, Witkin SS, Wohlgemuth SE, Wollert T, Wolvetang EJ, Wong E, Wong GW, Wong RW, Wong VKW, Woodcock EA, Wright KL, Wu C, Wu D, Wu GS, Wu J, Wu J, Wu M, Wu M, Wu S, Wu WKK, Wu Y, Wu Z, Xavier CPR, Xavier RJ, Xia GX, Xia T, Xia W, Xia Y, Xiao H, Xiao J, Xiao S, Xiao W, Xie CM, Xie Z, Xie Z, Xilouri M, Xiong Y, Xu C, Xu C, Xu F, Xu H, Xu H, Xu J, Xu J, Xu J, Xu L, Xu X, Xu Y, Xu Y, Xu ZX, Xu Z, Xue Y, Yamada T, Yamamoto A, Yamanaka K, Yamashina S, Yamashiro S, Yan B, Yan B, Yan X, Yan Z, Yanagi Y, Yang DS, Yang JM, Yang L, Yang M, Yang PM, Yang P, Yang Q, Yang W, Yang WY, Yang X, Yang Y, Yang Y, Yang Z, Yang Z, Yao MC, Yao PJ, Yao X, Yao Z, Yao Z, Yasui LS, Ye M, Yedvobnick B, Yeganeh B, Yeh ES, Yeyati PL, Yi F, Yi L, Yin XM, Yip CK, Yoo YM, Yoo YH, Yoon SY, Yoshida KI, Yoshimori T, Young KH, Yu H, Yu JJ, Yu JT, Yu J, Yu L, Yu WH, Yu XF, Yu Z, Yuan J, Yuan ZM, Yue BYJT, Yue J, Yue Z, Zacks DN, Zacksenhaus E, Zaffaroni N, Zaglia T, Zakeri Z, Zecchini V, Zeng J, Zeng M, Zeng Q, Zervos AS, Zhang DD, Zhang F, Zhang G, Zhang GC, Zhang H, Zhang H, Zhang H, Zhang H, Zhang J, Zhang J, Zhang J, Zhang J, Zhang JP, Zhang L, Zhang L, Zhang L, Zhang L, Zhang MY, Zhang X, Zhang XD, Zhang Y, Zhang Y, Zhang Y, Zhang Y, Zhang Y, Zhao M, Zhao WL, Zhao X, Zhao YG, Zhao Y, Zhao Y, Zhao YX, Zhao Z, Zhao ZJ, Zheng D, Zheng XL, Zheng X, Zhivotovsky B, Zhong Q, Zhou GZ, Zhou G, Zhou H, Zhou SF, Zhou XJ, Zhu H, Zhu H, Zhu WG, Zhu W, Zhu XF, Zhu Y, Zhuang SM, Zhuang X, Ziparo E, Zois CE, Zoladek T, Zong WX, Zorzano A, Zughaier SM (2016). Guidelines for the use and interpretation of assays for monitoring autophagy (3rd edition). Autophagy.

[23] Belenky P, Racette FG, Bogan KL, McClure JM, Smith JS, Brenner C (2007). Nicotinamide riboside promotes Sir2 silencing and extends lifespan via Nrk and Urh1/Pnp1/Meu1 pathways to NAD+. Cell.

[24] Wolf G (2006). Calorie restriction increases life span: a molecular mechanism. Nutr Rev.

[25] Imai S, Guarente L (2014). NAD+ and Sirtuins in aging and disease. Trends Cell Biol.

[26] Sadun AA, Smith LE, Kenyon KR (1983). Paraphenylenediamine: a new method for tracing human visual pathways. J Neuropathol Exp Neurol.

[27] Sase K, Kitaoka Y, Munemasa Y, Kojima K, Takagi H (2015). Axonal protection by short-term hyperglycemia with involvement of autophagy in TNF-induced optic nerve degeneration. Front Cell Neurosci.

[28] Kitaoka Y, Sase K, Tsukahara C, Fujita N, Tokuda N, Kogo J, Takagi H (2020). Axonal protection by a small molecule SIRT1 activator, SRT2104, with alteration of autophagy in TNF-induced optic nerve degeneration. Jpn J Ophthalmol.

[29] Schöndorf DC, Ivanyuk D, Baden P, Sanchez-Martinez A, De Cicco S, Yu C, Giunta I, Schwarz LK, Di Napoli G, Panagiotakopoulou V, Nestel S, Keatinge M, Pruszak J, Bandmann O, Heimrich B, Gasser T, Whitworth AJ, Deleidi M (2018). The NAD+ precursor nicotinamide riboside rescues mitochondrial defects and neuronal loss in iPSC and fly models of Parkinson's disease. Cell Rep.

[30] Xie X, Gao Y, Zeng M, Wang Y, Wei TF, Lu YB, Zhang WP (2019). Nicotinamide ribose ameliorates cognitive impairment of aged and Alzheimer's disease model mice. Metab Brain Dis.

[31] Harlan BA, Killoy KM, Pehar M, Liu L, Auwerx J, Vargas MR (2020). Evaluation of the NAD+ biosynthetic pathway in ALS patients and effect of modulating NAD+ levels in hSOD1-linked ALS mouse models. Exp Neurol.

[32] Vaur P, Brugg B, Mericskay M, Li Z, Schmidt MS, Vivien D, Orset C, Jacotot E, Brenner C, Duplus E (2017). Nicotinamide riboside, a form of vitamin B3, protects against excitotoxicity-induced axonal degeneration. FASEB J.

[33] Shen C, Dou X, Ma Y, Ma W, Li S, Song Z (2017). Nicotinamide protects hepatocytes against palmitate-induced lipotoxicity via SIRT1-dependent autophagy induction. Nutr Res.

[34] Salminen A, Kaarniranta K (2009). SIRT1 regulation of longevity via autophagy. Cell Signal.

[35] Luo G, Jian Z, Zhu Y, Zhu Y, Chen B, Ma R, Tang F, Xiao Y (2019). Sirt1 promotes autophagy and inhibits apoptosis to protect cardiomyocytes from hypoxic stress. Int J Mol Med.

[36] Imai S, Guarente L (2016). It takes two to tango: NAD+ and sirtuins in aging/longevity control. NPJ Aging Mech Dis.

[37] Joshi U, Evans JE, Pearson A, Saltiel N, Cseresznye A, Darcey T, Ojo J, Keegan AP, Oberlin S, Mouzon B, Paris D, Klimas N, Sullivan K, Mullan M, Crawford F, Abdullah L (2020). Targeting sirtuin activity with nicotinamide riboside reduces neuroinflammation in a GWI mouse model. Neurotoxicology..

[38] Hedya SA, Safar MM, Bahgat AK (2018). Cilostazol mediated Nurr1 and autophagy enhancement: neuroprotective activity in rat rotenone PD model. Mol Neurobiol.

[39] Fletcher RS, Lavery GG (2018). The emergence of the nicotinamide riboside kinases in the regulation of NAD+ metabolism. J Mol Endocrinol.

