# Supplementary figures and images for: Axonal Protection by Nicotinamide Riboside via SIRT1-Autophagy Pathway in TNF-Induced Optic Nerve Degeneration
Source: Mol Neurobiol. 2020 Aug 20;57(12):4952–60. doi: 10.1007/s12035-020-02063-5 (PMC7541376; doi:10.1007/s12035-020-02063-5)

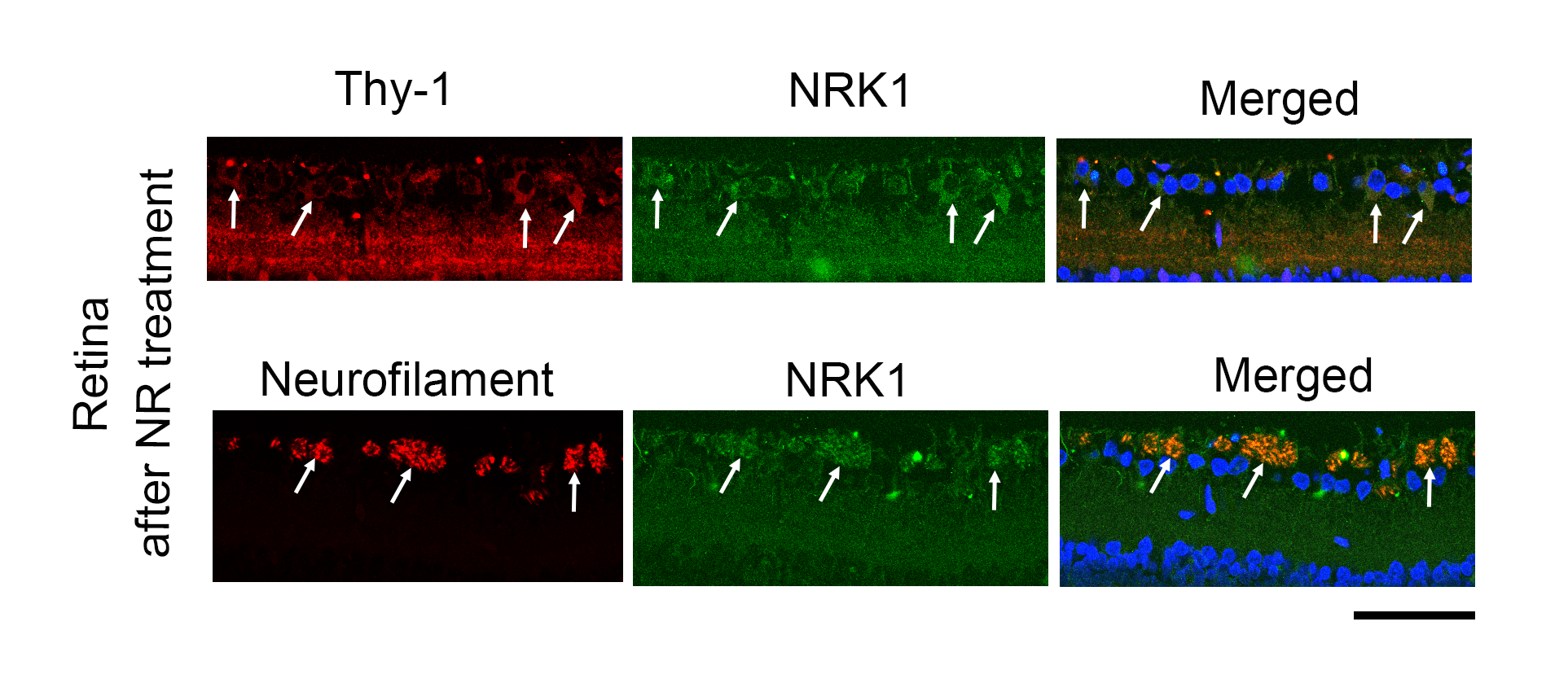

Supplement: Supplementary file 1 — Immunohistochemistry in the NR-treated retina. NRK1-positive cells were colocalized with Thy-1-positive cells. NRK1 immunoreactivity was colocalized with neurofilament immunoreactivity. Arrows indicate colocalization. Scale bar = 50 μm. (PNG 980 kb). [file 12035_2020_2063_Fig8_ESM.png]

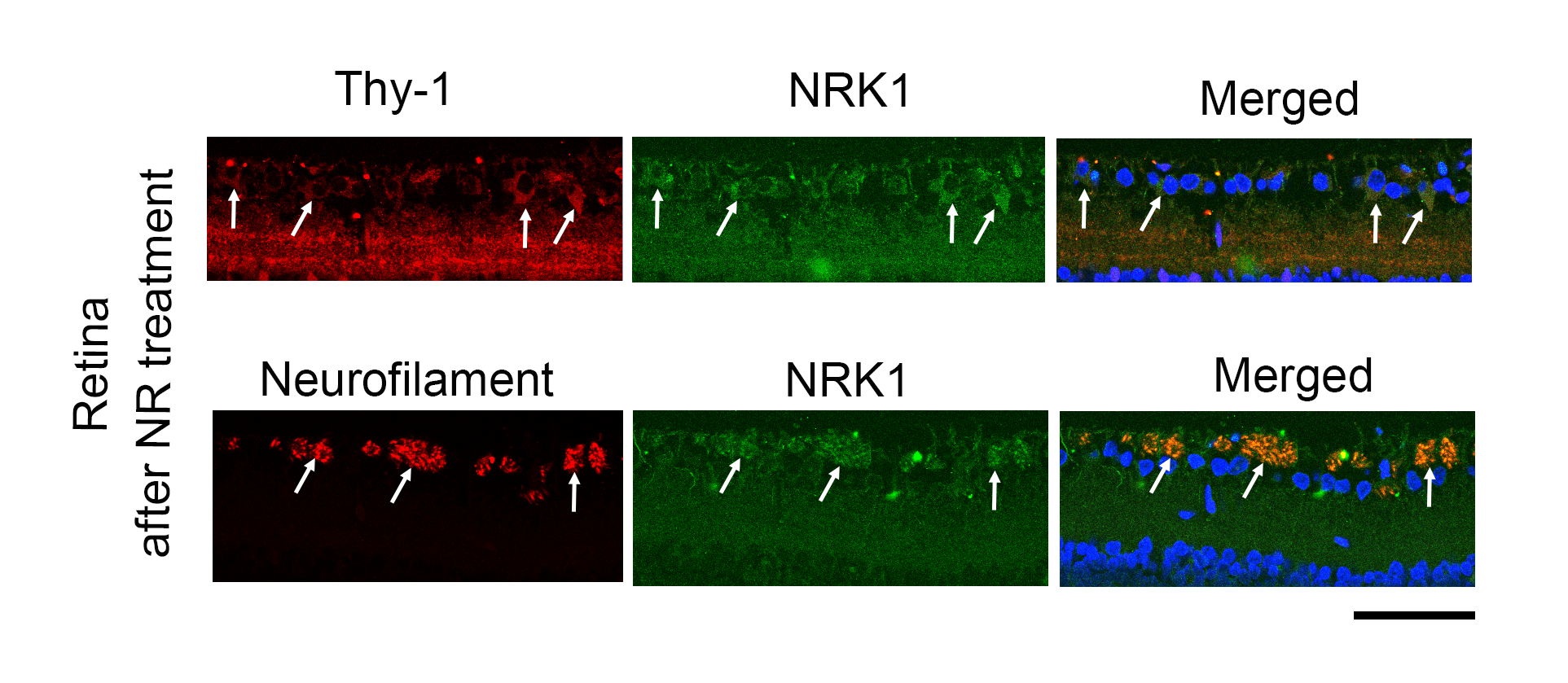

Supplement: Supplementary file 2 — High resolution image (TIF 4757 kb). [file 12035_2020_2063_MOESM1_ESM.tif]
